# Deletion of Protein Tyrosine Phosphatase 1B (PTP1B) Enhances Endothelial Cyclooxygenase 2 Expression and Protects Mice from Type 1 Diabetes-Induced Endothelial Dysfunction

**DOI:** 10.1371/journal.pone.0126866

**Published:** 2015-05-14

**Authors:** David J. Herre, J. Blake Norman, Ruchi Anderson, Michel L. Tremblay, Anne-Cecile Huby, Eric J. Belin de Chantemèle

**Affiliations:** 1 Physiology Department, Medical College of Georgia at Georgia Regents University, Augusta, GA, United States of America; 2 Goodman Cancer Center and Department of Biochemistry, McGill University, Montreal, QC, Canada; Goethe Universität Frankfurt, GERMANY

## Abstract

Protein tyrosine phosphatase 1B (PTP1B) dephosphorylates receptors tyrosine kinase and acts as a molecular brake on insulin signaling pathway. Conditions of metabolic dysfunction increase PTP1B, when deletion of PTP1B protects against metabolic disorders by increasing insulin signaling. Although vascular insulin signaling contributes to the control of glucose disposal, little is known regarding the direct role of PTP1B in the control of endothelial function. We hypothesized that metabolic dysfunctions increase PTP1B expression in endothelial cells and that PTP1B deletion prevents endothelial dysfunction in situation of diminished insulin secretion. Type I diabetes (T1DM) was induced in wild-type (WT) and PTP1B-deficient mice (KO) with streptozotocin (STZ) injection. After 28 days of T1DM, KO mice exhibited a similar reduction in body weight and plasma insulin levels and a comparable increase in glycemia (WT: 384±20 vs. Ko: 432±29 mg/dL), cholesterol and triglycerides, as WT mice. T1DM increased PTP1B expression and impaired endothelial NO-dependent relaxation, in mouse aorta. PTP1B deletion did not affect baseline endothelial function, but preserved endothelium-dependent relaxation, in T1DM mice. NO synthase inhibition with L-NAME abolished endothelial relaxation in control and T1DM WT mice, whereas L-NAME and the cyclooxygenases inhibitor indomethacin were required to abolish endothelium relaxation in T1DM KO mice. PTP1B deletion increased COX-2 expression and PGI_2_ levels, in mouse aorta and plasma respectively, in T1DM mice. In parallel, simulation of diabetic conditions increased PTP1B expression and knockdown of PTP1B increased COX-2 but not COX-1 expression, in primary human aortic endothelial cells. Taken together these data indicate that deletion of PTP1B protected endothelial function by compensating the reduction in NO bioavailability by increasing COX-2-mediated release of the vasodilator prostanoid PGI_2_, in T1DM mice.

## Introduction

In addition to its key role in the control of metabolic function and glucose disposal, insulin is also a main contributor to the maintenance of physiological endothelial function [1]. Vascular endothelial cells express insulin receptors and binding of insulin to the extracellular domain of the insulin receptor (IR) leads to its phosphorylation and to the consequent activation of the phosphatidylinositol 3-kinase (PI3K)-Akt signaling pathway, which induces phosphorylation and activation of the endothelial NO synthase (eNOS). The resulting secretion and release of nitric oxide (NO) promotes vascular relaxation, capillary recruitment, increases blood flow and inhibits vascular smooth muscle cell proliferation and leucocytes adhesion. This combination of effects facilitates glucose disposal and prevents vascular dysfunction [1].

Diminished production of insulin in type 1 diabetes (T1DM) or resistance to insulin action in type II diabetes mellitus (T2DM) not only leads to hyperglycemia, a major triggering factor of endothelial dysfunction, but also to a reduction in the vasculoprotective effects of insulin [2] which further enhance the deleterious effects of hyperglycemia on vascular function. In patients with diabetes, impaired endothelial function is a consistent finding [1, 2] and a leading cause of micro- and macrovascular complications causing disability and death in patients with diabetes and for which therapeutic strategies are still needed.

Insulin signaling is under the control of protein tyrosine phosphatases such as protein tyrosine phosphatase 1B (PTP1B) that interrupts insulin signaling by dephosphorylating both the insulin receptor and insulin receptor substrate. Conditions of metabolic dysfunction, such as obesity and diabetes, are associated with increased PTP1B expression in the brain and metabolic tissues [3–5], which provides a potential explanation for the development of insulin resistance. However, whether metabolic disorders are associated with increased PTP1B expression in endothelial cells remains unknown.

Genome-wide and tissue specific deletion of PTP1B increases insulin sensitivity and confers protection against obesity and type II diabetes in mice [6, 7]. Similarly, preservation of insulin sensitivity through PTP1B deletion or pharmacological inhibition of PTP1B protects against obesity- [8], hypertension- [9] and heart failure- [10] induced endothelial dysfunction. However, whether deletion of PTP1B represents a protective mechanism against endothelial dysfunction in situations of diminished insulin secretion remains unknown.

The goals of the present study are to reach a better understanding of the role of PTP1B in the control of endothelial function in the context of metabolic disease and to determine the viability of PTP1B as a therapeutic target. We hypothesized that diabetic conditions will increase PTP1B expression in the vasculature and that deletion of PTP1B is protective of endothelial function in the face of T1DM. We induced an insulin-deficient state in PTP1B knockout mice and mimicked diabetic conditions in cultured endothelial cells to determine the contribution of PTP1B in controlling endothelial function.

## Materials and Methods

### Animal Model

PTP1B null mice (KO) were generated on a Balb/c background at Goodman Cancer Center of McGill University [6, 9, 11] and compared to their wild-type control (WT). All animals were kept under conditions of constant temperature (22 ± 2°C) with a 12h light/12h dark cycle and were fed standard mouse chow. Tap water was provided ad libitum. Mice were housed in an American Association of Laboratory Animal Care approved animal care facility at Georgia Regents University. Georgia Regents University Institutional Animal Care and Use Committee approved all protocols.

### Streptozotocin Treatment

Type I diabetes was induced following the AMDCC Low Dose Streptozotocin Induction Protocol (https://www.diacomp.org/shared/protocols.aspx). Briefly, 10–12 week old male WT and PTP1B KO mice were divided into two groups and treated (*ip*. injection) with either vehicle (Na-citrate buffer) or Streptozotocin (STZ, 50mg/kg) for five consecutive days. Induction of type 1 diabetes was confirmed by glycemia greater than 240 mg/dl at seven days after the last injection.

### Metabolic profile

Blood glucose was assessed with a glucometer (Precision XL, Abbot Laboratories, Alameda, CA, USA) seven days after the last injection and at the time of euthanasia to ensure sufficient levels of hyperglycemia. Plasma insulin and leptin levels were determined using colorimetric assays from ALPCO Diagnostics (Salem, NH, USA). Plasma total cholesterol and triglycerides were assessed with colorimetric assays (Wako, Richmond, VA, USA).

### Vascular Reactivity

Twenty-eight days after STZ treatment, mice were anesthetized (isoflurane 5%) and euthanized according to the approved protocol. Trunk blood was collected and thoracic aorta were dissected surgically, cleaned of fat, cut in 2mm-long rings and mounted on a wire myograph (DMT, Aarhus, Denmark) with 1g of basal tension. Endothelial function was determined according to the protocol previously described [8, 9, 12]. Briefly, aortic rings' endothelial function was assessed with concentration-response curves to acetylcholine (ACh, 1 pmol/L to 10 μmol/L). Contribution of NO or cyclooxygenases (COXs) to vascular relaxation was determined by pre-incubation of the aortic rings with either the nonspecific NO synthase inhibitor N^ω^-nitro-L-arginine methyl ester (L-NAME; 100μmol/L), the COXs inhibitor Indomethacin (100μmol/L), or the combination of both L-NAME and Indomethacin. Endothelium-independent relaxation was analyzed with a concentration-response curve to sodium nitroprusside (SNP, 1pmol/L to 10μmol/L). Relaxation curves were performed on preconstricted vessels (5HT 0.1–1μmol/L) and relaxation was expressed as a percent of the precontraction. Area under the curve was calculated for each curve to determine the contribution of each treatment.

### Plasma Prostaglandin I_2_ levels

Indirect measurement of plasma PGI_2_ levels were obtained by measuring plasma levels of the PGI_2_ metabolite: 6-keto Prostaglandin F1α via (EIA kit, Cayman, Ann Arbor, MI, USA).

### Chronic systemic COX-2 inhibition

A subset of WT and PTP1B KO mice was chronically treated with the selective COX-2 inhibitor celecoxib (Toronto Research Chemical Inc.). Treatment was initiated immediately after the last STZ injection, by switching mice from a regular mouse chow to a regular chow supplemented in celecoxib (190mg/kg of chow) to reach a dose of 25mg/kg/day [13, 14].

### Endothelial Cell Culture and knock down of PTP1B via gene silencing

Human aortic endothelial cells (HAECs) were purchased from Lonza and grown (passage 3–9) in EBM2 medium containing 100 U/ml penicillin, 100 mg/ml streptomycin, and 10% FBS. PTP1B expression was repressed via viral infection of 90% confluent cells with either a control or PTP1B siRNA (Silencer Select siRNA PTPN1, Invitrogen, catalogue# 1105) in the presence of Lipofectamine RNAiMAX (Invitrogen). Cells were exposed to either a low (5 mM) or high glucose (25 mM) concentration for 5 days to mimic diabetic conditions. At the end of the experiments cells were harvested for Western-blot.

### Western Blot Analysis

Aortic tissue and HAECs homogenates (10 to 25 μg) were separated via SDS-PAGE and transferred to Immobilon-P poly(vinylidene fluoride) membranes. Immunoblots were probed with antibodies for PTP1B (mouse: Abcam; human: BD Bioscience), GAPDH (Santa Cruz), COX-1 (Abcam), COX-2 (BD Bioscience), endothelial NO synthase (BD Bioscience), and phosphorylated endothelial NO synthase 1177/79 (BD Bioscience), Akt, and phosphorylated Akt (Ser^473^, Cell Signaling).

### Statistical Analysis

All data are presented as mean±sem. Differences in means among groups for non-repeated variables were compared by 1-way ANOVA. Differences in means among groups and treatments, with repeated variables, were compared by 2- or 3-way ANOVA with repeated measures, when appropriate. Bonferroni and Fisher least significant difference tests were used as the post hoc test (Statview).

## Results

### Baseline Phenotype

Induction of Type I diabetes was confirmed by measuring insulin and blood glucose levels after 28 days of diabetes. As reported in Table 1, STZ treatment significantly reduced insulin levels and increased blood glucose in both WT and KO animals consistent with a T1DM state. Insulin levels that were initially reduced with PTP1B deletion further decreased with T1DM. T1DM induced a similar decline in body weight in WT and KO animals and had no effect on plasma leptin levels. Deletion of PTP1B did not affect plasma cholesterol and triglycerides levels, which were, however, similarly increased in WT and KO animals with T1DM.

**Table 1:** Basic Physiological Parameters.

	WT		KO	
	Control	STZ	Control	STZ
**Body Weight, g**	30±0.5	*26±0.5	31±1.2	*26±1.4
**Glycemia, mg/dL**	189±21	*384±20	172±12	*432±29
**Insulin, pg/mL**	409±23	*310±17	^#^325±16	* ^#^263±9
**Leptin, pg/mL**	811±161	616±50	497±59	633±176
**Cholesterol, mg/dL**	59±4	*112±8	64±4	*134±11
**Triglycerides, mg/dL**	30±2	*106±14	53±7	*81±13

Data are mean±SEM, n = 8 per group,

*p<0.05 *vs*. Control within the same strain,

^#^p<0.05 *vs*. WT.

### Endothelial Function

**Fig 1:**
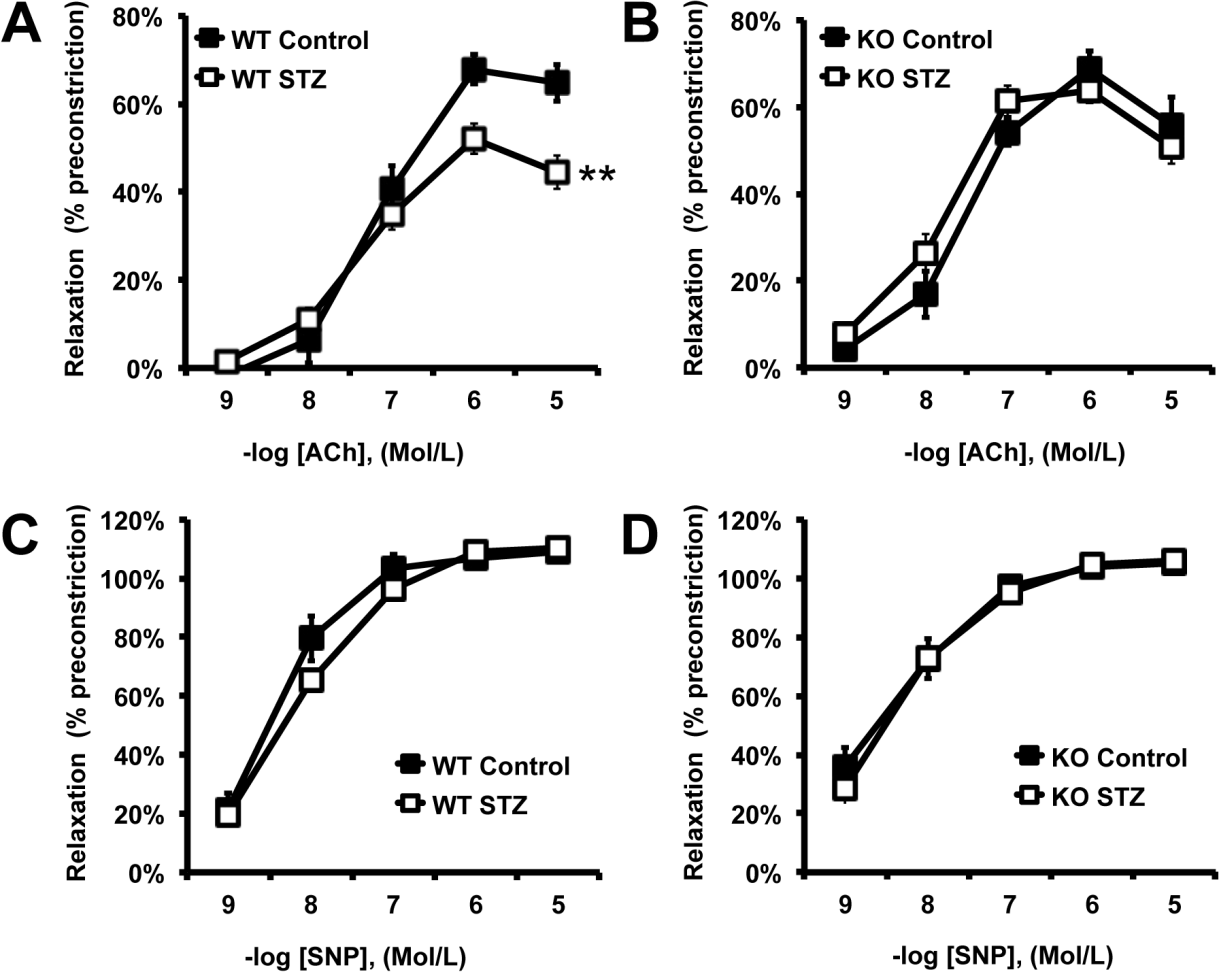
PTP1B deletion prevents STZ-induced endothelial dysfunction. Concentration response curves to acetylcholine (ACh, **A**, **B**)) and sodium nitroprusside (SNP, **C, D**) conducted in wild-type (WT) and PTP1B KO mice (KO) treated with citrate-buffer (control) or streptozotocin (STZ). Data are mean±SEM, n = 6 per group, **p<0.001 *vs*. Control.

**Fig 2:**
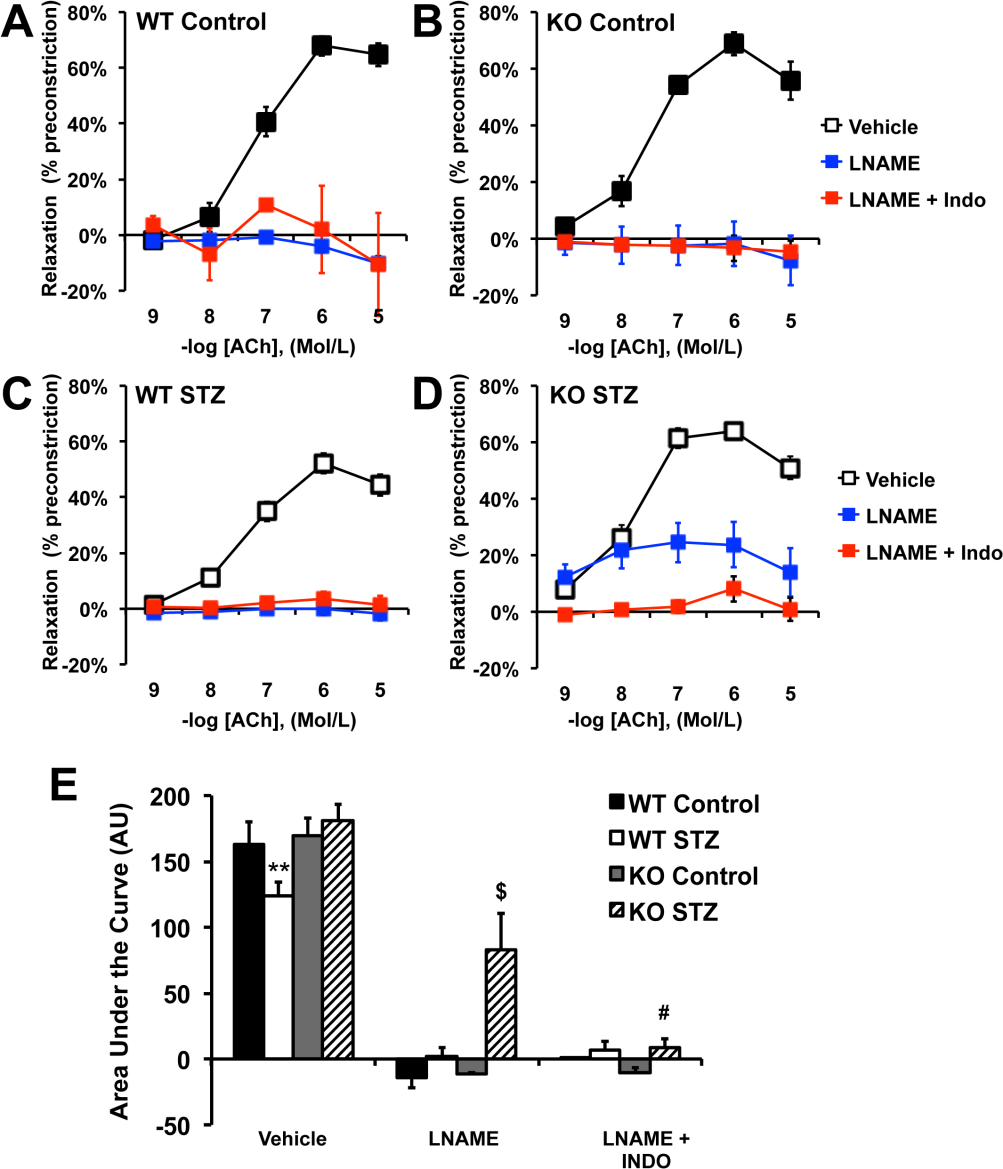
Cyclooxygenase derivatives preserve endothelial function in PTP1B KO mice treated with STZ. A-D: Concentration response curves to acetylcholine (ACh) conducted in the presence or not of the NOS inhibitor L-NAME (10μM) or the cyclooxygenases inhibitor indomethacin (Indo,10μM). E: Quantification of the area under the curve for each concentration response curve. Data are mean±SEM, n = 6 per group, **p<0.001 *vs*. Control within the same group, ^$^p<0.05 *vs*. vehicle, ^#^p<0.05 *vs*. L-NAME.

**Fig 3:**
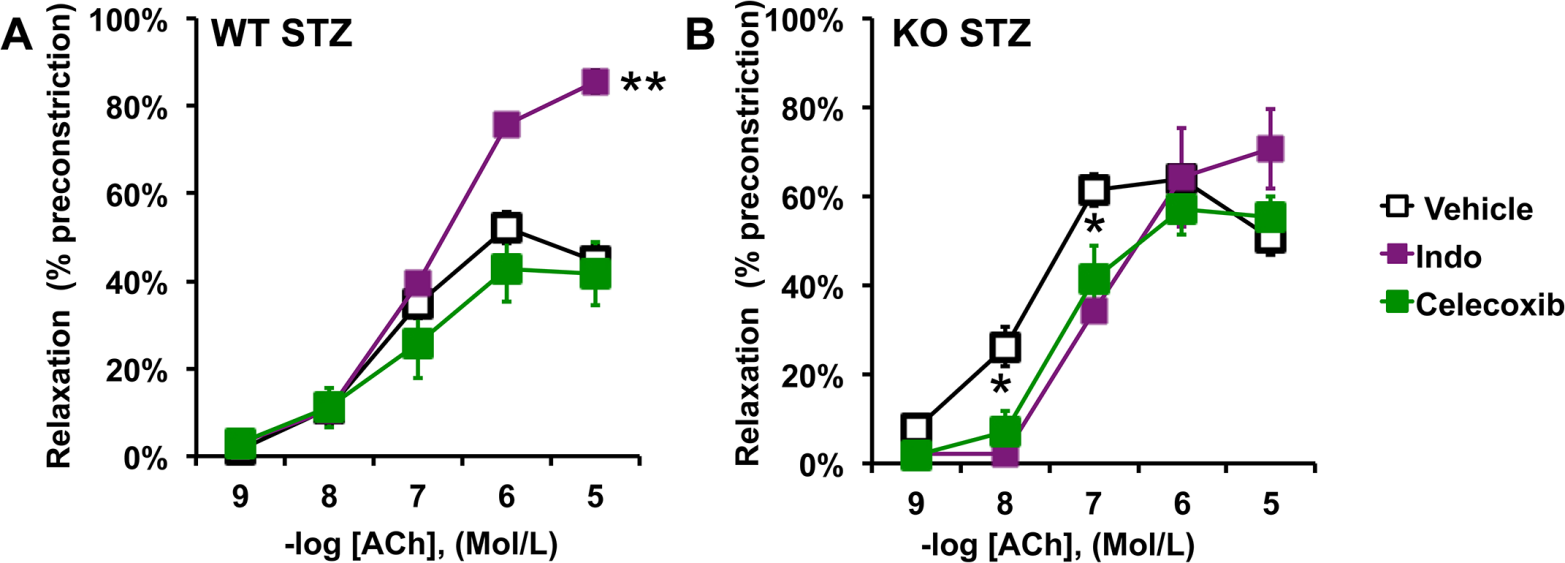
Acute or chronic COX-2 inhibition reduces endothelial sensitivity to ACh in PTP1B KO mice treated with STZ. Concentration response curves to acetylcholine (ACh) conducted in the presence or not of the unspecific cyclooxygenases inhibitor indomethacin (Indo,10μM) or in mice chronically treated with the selective COX-2 inhibitor, celecoxib (25mg/kg/day). Data are mean±SEM, n = 6 per group, *p<0.05, **p<0.001 *vs*. Vehicle within the same group.

We determined the effects of T1DM on endothelial function by measuring ACh-mediated relaxation of aortic rings. As reported in Fig 1, T1DM induced a significant reduction in ACh-mediated relaxation in WT animals. Consistent with our previous work, PTP1B deletion, *per se*, did not affect ACh-mediated relaxation [9] but prevented STZ-induced endothelial dysfunction (Fig 1B). T1DM did not affect endothelium-independent relaxation (SNP-mediated relaxation) in either WT or KO mice (Fig 1C and 1D), suggesting that the dysfunction observed is at the level of the endothelium. To identify the protective mechanisms developed by the PTP1B KO mice, concentration response curves to ACh were repeated in the presence of the NOS inhibitor L-NAME, with and without the cyclooxygenases (COXs) inhibitor indomethacin. As reported in Fig 2A, 2C and 2E, L-NAME completely abolished vascular relaxation in WT mice treated or not with STZ and also in control PTP1B KO mice (Fig 2B and 2E). These data indicate that the relaxation is fully dependent on NO in these animals and confirm that T1DM induces endothelial dysfunction by reducing NO bioavailability. Interestingly, L-NAME modestly reduced endothelium relaxation in PTP1B KO mice treated with STZ, whereas L-NAME+Indo completely abolished the relaxation (Fig 2D and 2E). This suggests that preservation of endothelial function involves vasodilator COXs derivatives in KO+STZ. The contribution of vasodilator prostanoids was further studied by repeating concentration response curves to ACh in the presence of indomethacin alone and in animals chronically treated with the selective COX-2 inhibitor, celecoxib. As reported in Fig 3A, acute COXs inhibition with indomethacin restored ACh-mediated relaxation, in WT+STZ. However, chronic celecoxib treatment did not improve endothelial function in these animals (Fig 3A). Together these data suggest that T1DM promotes COX-1-mediated release of vasoconstrictor prostanoids and that T1DM-induced endothelial dysfunction does not involve COX-2, in WT animals.

Both indomethacin and celecoxib reduced aortic ring sensitivity to ACh (shift of the curve towards high concentrations), in KO+STZ (Fig 3B). These data support the results gathered in the presence of L-NAME, which indicate that PTP1B deletion likely protects endothelial function by increasing the contribution of dilatory prostanoids to endothelium-dependent relaxation.

### Cyclooxygenase derivatives

**Fig 4:**
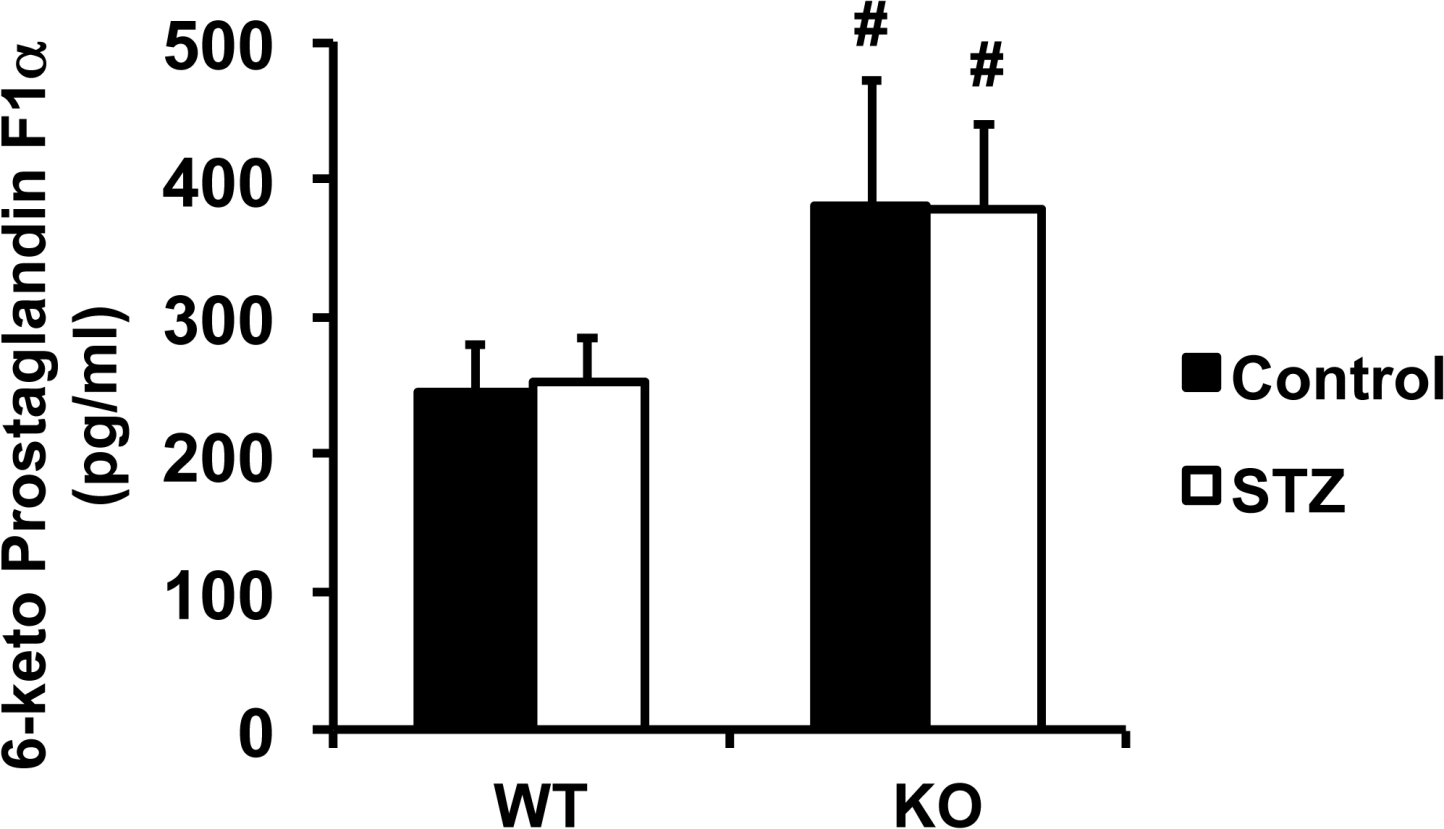
PTP1B deletion increased plasma 6-keto Prostaglandin F1α. Plasma levels of 6-keto Prostaglandin F1α. Data are mean±SEM, n = 8 per group. ^#^p<0.05 *vs*. WT.

To determine the potential contribution of COXs derivatives in the preservation of endothelial function in KO+STZ, we quantified plasma 6-keto Prostaglandin F1α levels. As reported in Figs 4 and 6-keto Prostaglandin F1α levels were significantly increased in PTP1B KO mice compared to WT. No changes in 6-keto Prostaglandin F1α levels were observed with diabetes in either WT or KO animals.

### Molecular Mechanisms

**Fig 5:**
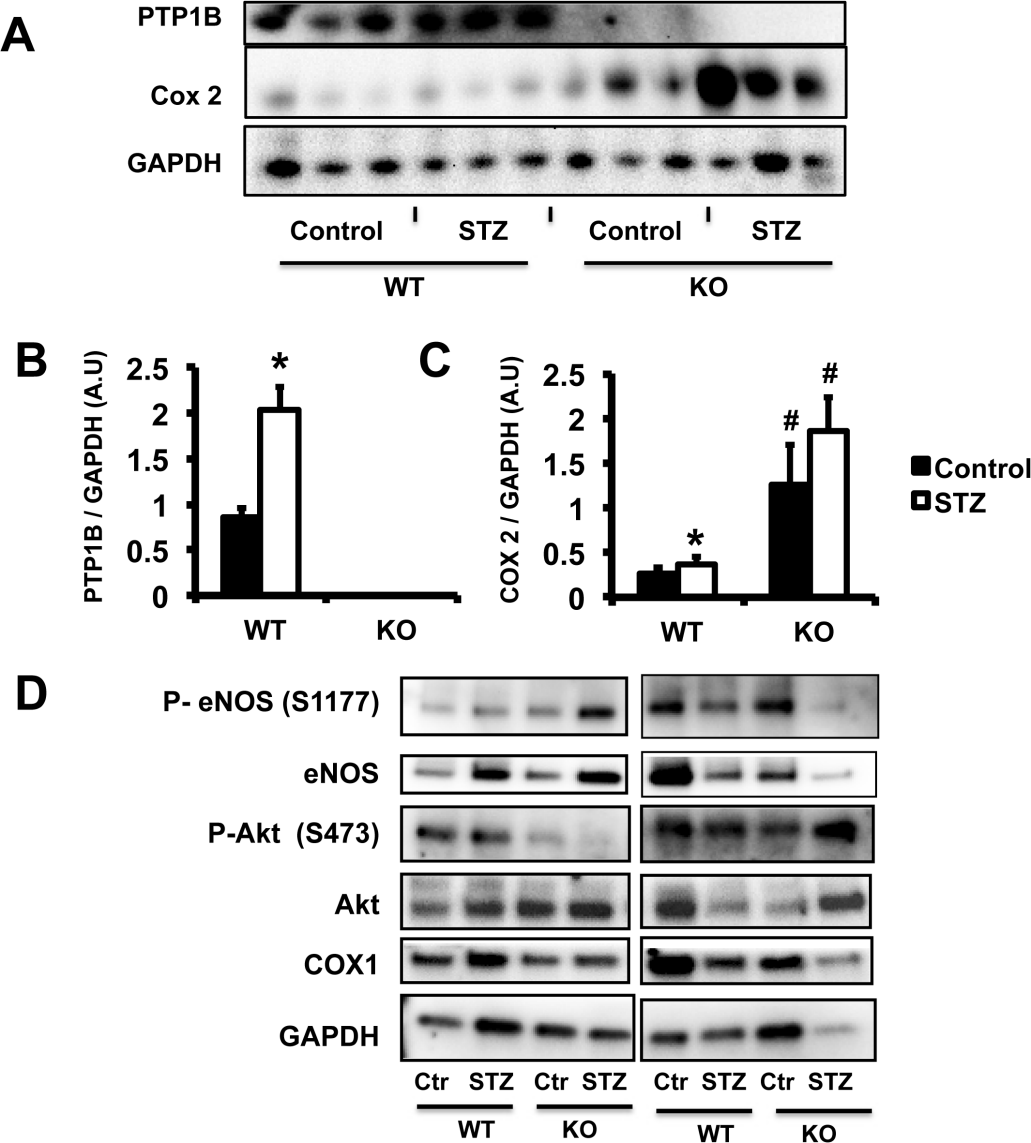
Diabetes increased PTP1B and COX-2 expression in aortic tissue. Quantification of aortic proteins expression via western-blot analysis. A, D: representative picture and B, C: densitometry. Data are mean±SEM, n = 6 per group. *p<0.05 *vs*. Control within the same group, ^#^p<0.05 *vs*. WT.

**Fig 6:**
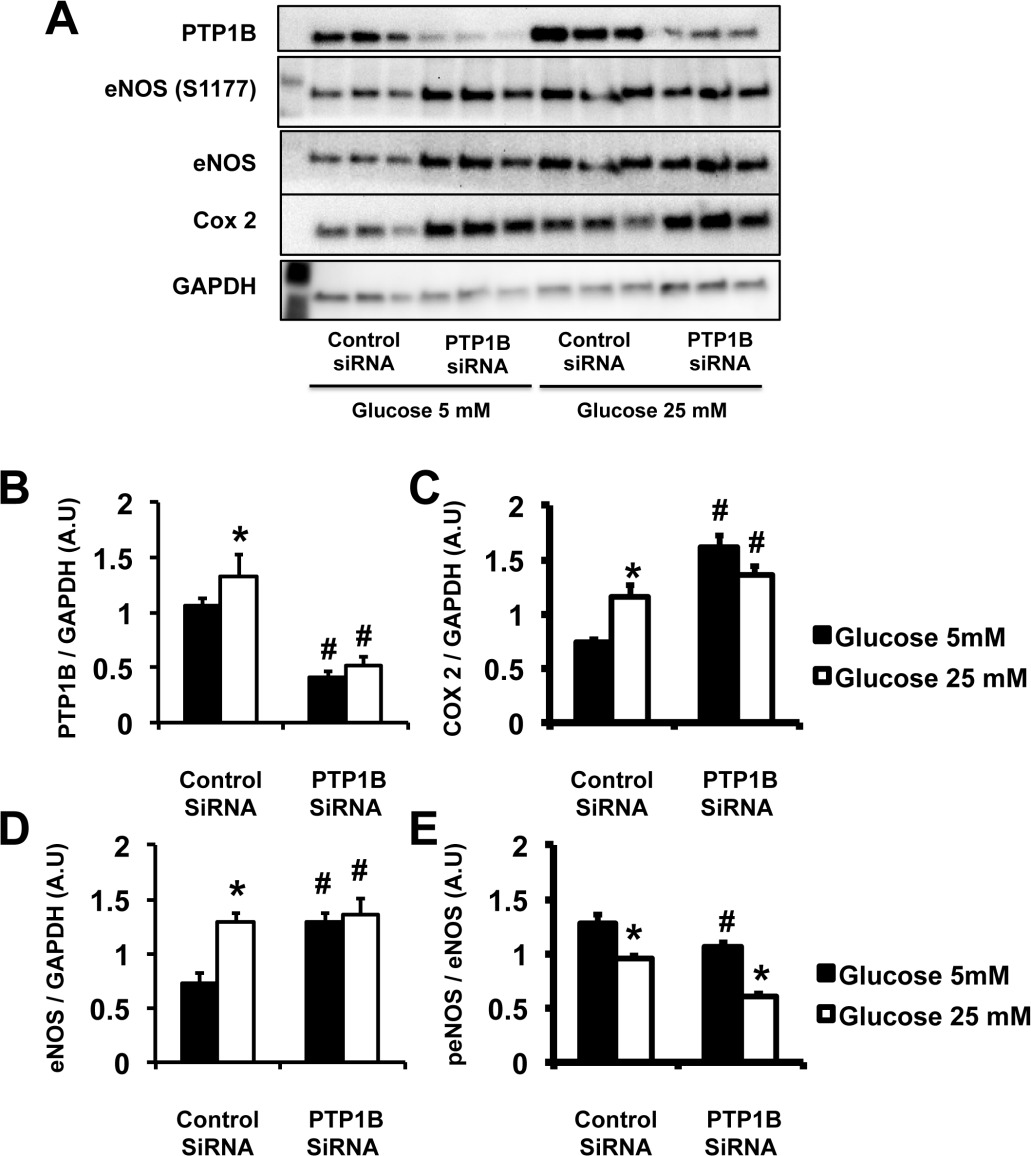
Diabetic conditions increased PTP1B and COX-2 expression in primary human endothelial cells. Quantification of proteins expression via western-blot analysis. A representative picture and B to E: densitometry. Data are mean±SEM, n = 3 per group, experiment repeated 3 times. *p<0.05 *vs*. Glucose 5mM, ^#^p<0.05 *vs*. Control siRNA.

To analyze whether PTP1B contributes to the control of endothelial function we quantified the level of expression of PTP1B in diabetic conditions. We reported that T1DM induced a significant increase in PTP1B expression in aortic rings of WT mice (Fig 5A and 5B). Similarly, five-day exposure to high glucose increased PTP1B expression in HAECs (Fig 6A and 6B).

Indomethacin abolished the protective effects of PTP1B deletion on endothelial function. Therefore, we measured the levels of expression of cyclooxygenases in aortic tissue and HAECs. We demonstrated that genome-wide deletion of PTP1B and 75% repression of PTP1B expression via siRNA increased COX-2 expression in aortic tissue (Fig 5A and 5C) and endothelial cells (Fig 6A and 6C), respectively. Diabetic conditions increased COX-2 expression in control HAECs but did not further increase COX-2 levels in aortic rings (Fig 5C) of PTP1B KO mice nor in HAECs infected with PTP1B siRNA (Fig 6C). We did not report any change in COX-1 expression with PTP1B deletion, diabetes, or exposure to high glucose concentration.

The effects of PTP1B deletion and diabetic conditions on Akt and eNOS were assessed by measuring their respective levels of expression and phosphorylation by western-blot analysis. PTP1B deletion and diabetic conditions did not affect the level of expression or phosphorylation of Akt and eNOS in mouse aortic tissue. High glucose exposure and PTP1B deletion increased eNOS expression in HAECs. However, high glucose did not further increase eNOS expression in HAECs deficient in PTP1B. High glucose exposure and PTP1B deletion reduced eNOS activity (ratio eNOS^S1177^/eNOS, Fig 5E), in HAECs. eNOS activity was further reduced in the HAECs deficient in PTP1B and exposed to high glucose (Fig 6E).

## Discussion

The goal of the present study was to analyze the role of PTP1B in the control of endothelial function and to determine whether PTP1B deletion could prevent endothelial dysfunction in situations of diminished insulin secretion. We reported for the first time that diabetic conditions increased PTP1B expression in endothelial cells and that genetic deletion of PTP1B protected animals from endothelial dysfunction by increasing COX-2 expression and PGI_2_ levels.

PTP1B is a molecular brake on insulin signaling. Increase in PTP1B expression or activity provides a mechanistic explanation for insulin resistance in the brain and metabolically active tissues (adipose tissue, liver, muscle, and hypothalamus) [3–5, 15–17] in situations of metabolic disorders such as obesity and diabetes. PTP1B is not confined to metabolically active tissues but rather is a ubiquitously expressed protein present in all vascular cell types [10, 18]. Interestingly, endothelial cells that play a minor role in the control of metabolic function exhibit a higher PTP1B expression than myocytes, adipocytes, or hepatocytes [19], which might explain the recently unraveled contribution of PTP1B in the control of endothelial function. Indeed, recent reports demonstrated that endothelial PTP1B controls vascular tree formation through the regulation of VEGRF2 signaling [5, 20]. Similarly, endothelial PTP1B has also been reported to contribute to adhesion molecule signaling and to regulate vascular inflammation and eosinophils recruitment [21]. However, whether metabolic disorders impair endothelial PTP1B expression and whether a change in PTP1B expression could affect endothelial function had not yet been studied. The present study demonstrated for the first time that diabetic conditions, even for a relative short period of time (28 days), increased PTP1B expression in mouse aorta. To determine whether metabolic disorders specifically increase PTP1B expression in endothelial cells, we used an *in vitro* approach and mimicked diabetic conditions in human aortic endothelial cells by exposing them to high glucose concentration for 5 days. Using this approach, we demonstrated that the increased in PTP1B expression, in mouse aorta, in response to diabetes, could be attributed to the endothelium. These data present hyperglycemia as a factor regulating PTP1B expression, but do, however, not rule out a potential additional contribution of cholesterol and triglycerides to the increase in PTP1B expression and the development of the endothelial dysfunction. While previous reports minimized the contribution of elevated lipids to diabetes-induced, COX-mediated endothelial dysfunction [22], no study analyzed the role of elevated lipids in the control of PTP1B expression. Further studies are consequently needed to determine whether diabetes-induced high triglycerides and cholesterol could also affect PTP1B expression.

Increased PTP1B expression in metabolically active tissues accompanied metabolic disorders but whether a similar increase in PTP1B expression in endothelial cells impairs endothelial function remained unknown. We addressed this question by measuring endothelium-dependent relaxation in aortic rings of mice submitted to T1DM for 28 days. The enhanced PTP1B expression observed in WT mice treated with STZ was associated with an impaired endothelium-dependent relaxation. Endothelium-independent relaxation remained intact. We analyzed the potential role of PTP1B in the reduced endothelium-dependent relaxation by repeating the same experiment in mice deficient in PTP1B and reported that PTP1B KO mice did not develop endothelial dysfunction in response to 28 days of diabetes. This suggested a direct role for PTP1B in the control of endothelial relaxation. A similar role for PTP1B has previously been suggested by the group of *Vincent Richard* who demonstrated that deletion or pharmacological inhibition of PTP1B prevented heart failure- or sepsis-induced endothelial dysfunction [10, 23]. The authors of the latter studies argued for a direct beneficial effect of PTP1B inhibitors on endothelial function. The potential contribution of an improved metabolic function was, however, not ruled out. In the present study, we reported that PTP1B deletion did not improve STZ-induced metabolic dysfunction. WT and PTP1B KO mice exhibited similar drops in body weight, and similar increases in blood glucose, cholesterol, and triglycerides levels in response to STZ. Although baseline insulin levels were significantly reduced by PTP1B deletion, WT and KO mice presented a similar decrease in insulin in response to STZ. Therefore, consistent with the conclusions of *Vercauteren et al*. [10], our data suggest that the vascular protective effects of PTP1B deletion are not mediated by an improved metabolic function but rather by a direct effect of PTP1B on endothelial cells.

NO is a main mediator of vascular relaxation in conductance arteries such as mouse aorta. In the present study, we demonstrated that NOS inhibition with L-NAME completely abolished relaxation, in both control and diabetic WT mice, confirming the NO-dependency of the relaxation and also the contribution of reduced NO bioavailability in the endothelial dysfunction associated with T1DM [22, 24–26]. In agreement with the literature [27], we could not explain the reduction in NO bioavailability by a low eNOS expression or activity. The well-characterized increases in reactive oxygen species [28–32] and constrictor prostanoids induced by T1DM (Fig 3A) are very likely the source of the dysfunction [22, 33].

Deletion of PTP1B did not alter the NO-dependency of the vascular relaxation. Indeed, as observed in WT animals, L-NAME completely abolished relaxation in PTP1B KO mice. L-NAME, however, barely reduced endothelium-dependent relaxation in PTP1B KO mice treated with STZ while the nonspecific COXs inhibitor, indomethacin, completely abolished endothelium-dependent relaxation. This lack of relaxation in the presence of indomethacin implies the contribution of vasodilator prostanoids. A role for dilatory prostanoids was further confirmed by demonstrating that acute (indomethacin) or chronic COX-2 inhibition (celecoxib) reduced aortic rings sensitivity to ACh, in diabetic PTP1B KO mice only. Consistent with these data, we reported increases in plasma prostacyclin levels and aortic COX-2 expression, in PTP1B KO mice. Similarly, PTP1B gene silencing increased COX-2 expression in HAECs. Thus, the increase in COX-2 expression observed in mouse aorta most likely reflects an increase in endothelial COX-2. Surprisingly, diabetes and hyperglycemia did not further raise plasma PGI_2_ or COX-2 expression in PTP1B KO mice and HAECs deficient in PTP1B. No clear mechanistic explanation could be provided. Nevertheless, it can be speculated that a phenomenon of saturation occurred. Collectively, these data suggest that release of PGI_2_ by endothelial COX-2 compensate for diabetes-induced reduction in NO bioavailability, in PTP1B KO mice treated with STZ. These data are in agreement with several reports from our group and others demonstrating that PGI_2_ compensate for reduced NO bioavailability, in hypertensive and diabetic patients [34, 35] in eNOS KO mice [36, 37], and in diabetic rodents [13, 38, 39]. The concept that COX-2 induction in endothelial cells represents an important compensatory mechanism to defend against vascular injury [40] is supported by the fact that endothelial cells constitutively express prostacyclin synthase which catalyzes the conversion of prostaglandin H_2_ to the vasodilator prostanoid: prostacyclin (PGI_2_) [40, 41]. In contrast, monocyte-macrophages, and fibroblasts do not express prostacyclin synthase and induced COX-2 expression in these cells results in increased synthesis of PGE_2_ and TXA_2_, which mediate inflammatory change, vasoconstriction, and platelet aggregation. This tissue-specific expression of prostaglandin synthase enzymes likely provides an explanation for the discrepancy between our study and the work by *Traves et al*., demonstrating that PTP1B deletion exaggerates LPS-induced COX-2 expression and promotes an inflammatory phenotype in macrophages [42]. However, the mechanisms whereby the deletion of PTP1B leads to an increase in COX-2 expression remain unknown. Insulin like growth factor-1 (IGF-1) regulates COX-2 but not COX-1 expression via PI3K signaling pathway [43]. As IGF-1 receptor activation is under the control of PTP1B, we could speculate that PTP1B deletion promotes IGF-1-induced COX-2 expression. However, further work is required to determine how PTP1B controls COX-2 expression.

Although increases in COX-2 expression and PGI_2_ appear as the most likely mechanisms by which PTP1B deletion protects the vascular endothelium, our data do, however, not rule out a direct role of PTP1B deletion on oxidative stress, the main source of reduced NO bioavailability in diabetes [28–32]. A direct role for PTP1B in the control of oxidative stress seems nevertheless unlikely. Indeed, although previous data from our group reported reduced oxidative stress levels in mesenteric arteries of obese mice deficient in PTP1B, these effects were the indirect consequence of an improved metabolic function and not a direct effect of PTP1B deletion on endothelial cell function [8]. Furthermore, ROS regulate PTP1B expression [44] and activity [45, 46], This rather places PTP1B downstream than upstream ROS.

In conclusion, the present study demonstrated for the first time that hyperglycemia-induced PTP1B expression in endothelial cells is associated with an impaired endothelial function and that deletion of PTP1B prevents diabetes-induced endothelial dysfunction via increased COX-2 derived PGI_2_. These data suggest that PTP1B inhibition might represent a novel therapeutic avenue for the treatment of diabetes associated endothelial dysfunction and might prevent diabetic macrovascular complications such as atherosclerosis and coronary heart disease.
