# Correction: Deletion of Protein Tyrosine Phosphatase 1B (PTP1B) Enhances Endothelial Cyclooxygenase 2 Expression and Protects Mice from Type 1 Diabetes-Induced Endothelial Dysfunction

**DOI:** 10.1371/journal.pone.0130781

**Published:** 2015-06-11

**Authors:** The PLOS ONE Staff

The first author’s name is spelled incorrectly. The correct name is: David J. Herren. The correct citation is: Herren DJ, Norman JB, Anderson R, Tremblay ML, Huby A-C, Belin de Chantemèle EJ (2015) Deletion of Protein Tyrosine Phosphatase 1B (PTP1B) Enhances Endothelial Cyclooxygenase 2 Expression and Protects Mice from Type 1 Diabetes-Induced Endothelial Dysfunction. PLoS ONE 10(5): e0126866. doi:10.1371/journal.pone.0126866

The publisher apologizes for this error, which was introduced during the typesetting process.
